# Amelioration of Dextran Sodium Sulfate-Induced Colitis in Mice through Oral Administration of Palmitoylethanolamide

**DOI:** 10.3390/biomedicines12051000

**Published:** 2024-05-02

**Authors:** Purvi Trivedi, Tanya Myers, Bithika Ray, Matthew Allain, Juan Zhou, Melanie Kelly, Christian Lehmann

**Affiliations:** 1 Department of Pharmacology, Dalhousie University, Halifax, NS B3H 4R2, Canada; purvi.trivedi@dal.ca (P.T.); tanya.myers@nshealth.ca (T.M.); bithika.ray@nshealth.ca (B.R.); mallain@panagrx.com (M.A.); melanie.kelly@dal.ca (M.K.); 2 Department of Anesthesia, Pain Management, and Perioperative Medicine, Dalhousie University, Halifax, NS B3H 4R2, Canada; juan.zhou@dal.ca; 3 Department of Ophthalmology, Dalhousie University, Halifax, NS B3H 4R2, Canada; 4 Department of Physiology and Biophysics, Dalhousie University, Halifax, NS B3H 4R2, Canada; 5 Department of Microbiology and Immunology, Dalhousie University, Halifax, NS B3H 4R2, Canada; 6 Department of Computer Science, Dalhousie University, Halifax, NS B3H 4R2, Canada

**Keywords:** palmitoylethanolamide, ulcerative colitis, anti-inflammatory, DSS-induced colitis

## Abstract

Inflammatory bowel disease (IBD) is a group of chronic disorders characterized by pain, ulceration, and the inflammation of the gastrointestinal tract (GIT) and categorized into two major subtypes: ulcerative colitis (UC) and Crohn’s disease. The inflammation in UC is typically restricted to the mucosal surface, beginning in the rectum and extending through the entire colon. UC patients typically show increased levels of pro-inflammatory cytokines, leading to intestinal epithelial apoptosis and mucosal inflammation, which impair barrier integrity. Chronic inflammation is associated with the rapid recruitment and inappropriate retention of leukocytes at the site of inflammation, further amplifying the inflammation. While UC can be managed using a number of treatments, these drugs are expensive and cause unwanted side effects. Therefore, a safe and effective treatment for UC patients is needed. Palmitoylethanolamide (PEA) is an endogenous fatty acid amide and an analog of the endocannabinoid anandamine. PEA administration has been found to normalize intestinal GIT motility and reduce injury in rodents and humans. In the current study, we examined the efficacy of PEA encapsulated in phytosomes following oral administration in experimental ulcerative colitis. Here, we showed that PEA at a human-equivalent dose of 123 mg/kg (OD or BID) attenuated DSS-induced experimental colitis as represented by the reduction in clinical signs of colitis, reduction in gross mucosal injury, and suppression of leukocyte recruitment at inflamed venules. These findings add to the growing body of data demonstrating the beneficial effects of PEA to control the acute phase of intestinal inflammation occurring during UC.

## 1. Introduction

Inflammatory bowel diseases (IBDs), including ulcerative colitis (UC) and Crohn’s disease, are chronic and recurrent disorders of the gastrointestinal tract [1]. They are characterized by intestinal inflammation resulting from the infiltration of neutrophils, lymphocytes, or macrophages and leading to mucosal disruption and ulceration [2]. Despite some shared characteristics, the two forms of IBD can be distinguished by differences in genetic predisposition, risk factors, and clinical, endoscopic, and histological features [3]. Inflammation in UC is characteristically restricted to the mucosal surface. It starts in the rectum and generally extends proximally in a continuous manner through the entire colon; however, some patients with proctitis or left-sided colitis might have a caecal patch of inflammation [4].

Although the exact pathogenesis of IBDs is poorly understood, there is evidence that it involves the interaction of the immune system, genetic susceptibility, and the environment. Secreted pro-inflammatory cytokines (e.g., TNF-α and IL-6), as well as reactive oxygen species generated by recruited immune cells such as neutrophils, cause intestinal epithelial apoptosis and mucosal inflammation, resulting in the loss of barrier integrity in patients with UC and IBD [5]. Furthermore, an increase in leukocyte recruitment is a key feature of colitis and is regulated by the interaction between endothelial adhesion molecules and their specific ligands on leukocytes [6,7]. Importantly, chronic inflammation is associated with the rapid recruitment and often inappropriate retention of leukocytes at the site of inflammation, which likely amplifies the inflammatory response [8]. Therefore, the repair of the mucosal barrier and suppression of inflammation are effective strategies for the treatment of UC. Conventional drugs for UC include corticosteroids, amino salicylates, and antibiotics, as well as anti-TNF-α therapies [3,6,9]. However, these drugs have limitations including substantial economic burden and undesirable side-effects, e.g., anti-TNF-α medications can cause malignancies, infections, and congestive heart failure [10]. Hence, an effective and safe treatment for UC patients is urgently needed.

Palmitoylethanolamide (PEA) is an endogenous amide belonging to the family of fatty acid ethanolamides (FAEs) [11]. The anti-inflammatory and analgesic effects of PEA are likely mediated by direct or indirect targeting on a number of receptors, such as cannabinoid type 1 receptor (CB1) and cannabinoid type 2 receptor (CB2) in breast cancer cells [12], transient receptor potential vanilloid type-1 (TRPV1) ion channels in sensory neurons [13], and PPARα [14,15] and the orphan receptor GPR55 in striatal neurons [16]. PEA has been detected in the rodent and human digestive tracts, and when administered i.p., it normalizes intestinal motility [17] and reduces intestinal injury caused by ischemia–reperfusion [14]. PEA confers this protection on intestinal injury partially through its effect on the PPARα receptor [14]. However, whether PEA exhibits any protective effects in relieving clinical signs of ulcerative colitis in mice is not known.

In the present study, we employed a phytosomal drug delivery system to administer PEA through oral gavage in mice. The treatment represents a novel pharmacological approach utilizing the encapsulation of PEA in phytosomes in order to improve oral absorption. We aimed to evaluate the effects of PEA in a mouse model of dextran sodium sulphate (DSS)-induced colitis. We investigated whether PEA treatment attenuates DSS-induced clinical signs of colitis, pathological alterations of the colonic structure, and colonic leukocyte recruitment in mice. 

## 2. Materials and Methods

### 2.1. Animals

Male C57BL/6 mice were purchased from Charles River Laboratories International Inc. (Saint-Constant, QC, Canada). All mice were of wild type and 12–14 weeks old, with 20–30 g body weight, and housed in ventilated plastic cage racks in a pathogen-free room at the Carleton Animal Care Facility (CACF), Faculty of Medicine, Dalhousie University, Halifax, NS, Canada. Animals were kept on a 12 h light/dark cycle at 21 °C and were acclimatized for one week prior to experimentation. All experimental procedures were approved by the University Committee on Laboratory Animals at Dalhousie University under protocol number #21-113 and were performed following the guidelines and standards of the Canadian Council on Animal Care. 

### 2.2. Experimental Model 

Experimental colitis was induced via administration of 4% or 5% DSS (*w*/*v*) in drinking water (36–50 kDa; Cat # 160110, MP Biomedicals, Solon, OH, USA) for five days. On day 6, DSS water was replaced with fresh drinking water for one day. Control mice received only normal drinking water for the entire course of the experiment. Over the six-day study period, mice were monitored daily for body weight, their overall health (eye opening, fur and motor activity), stool consistency, and blood in the stool to obtain a clinical illness score (Table 1). On the morning of day seven, mice were sacrificed and colon was excised. Subsequently, colon was placed on filter paper to measure its length and weight was recorded. The colon was then processed for histological analysis.

#### 2.2.1. PEA Phytosome Preparation

Based on the dose of PEA employed in clinical trials, human-equivalent dose of PEA was chosen (123 mg/kg) once daily (OD) or twice daily (BID), p.o. PEA phytosome composition contains 40% PEA/60% excipients (50% sunflower lecithin, 8% microcrystalline cellulose, and 2% silicon dioxide). PEA phytosome was prepared by reconstituting 30.7 mg of PEA phytosome to 5 mL purified water to make a stock solution of 24.6 mg/mL PEA and 36.9 mg/mL excipients. Placebo phytosome or empty phytosome was prepared by reconstituting 184.5 mg of placebo phytosome to 5 mL purified water to make a stock solution of 36.9 mg/mL placebo excipient. Both placebo phytosome and PEA phytosome have the same concentration of excipients per dosing volume. The dosing volume of 10 mL/kg was used. 

#### 2.2.2. Experimental Groups

Mice were divided into following groups (*n* = 14/group):

1. Water + no treatment;
2. DSS + no treatment;
3. Water + PEA (123 mg/kg, p.o., OD);
4. Water + PEA (123 mg/kg, p.o., BID);
5. DSS + PEA (123 mg/kg, p.o., OD);
6. DSS + PEA (123 mg/kg, p.o., BID).

Experiments in groups 3–10 were repeated with empty phytosome gavage.

### 2.3. Tissue Processing and Histology

Briefly, paraffin tissue slices (thickness, 5 μm) were deparaffinized with xylene, stained with hematoxylin and eosin (H&E), and observed through light microscopy (Axio Vision, Zeiss, Milan, Italy). The degree of inflammation on microscopic cross-sections of the colon was graded semiquantitatively from 0 to 4 as previously described by [18,19]; in particular, the morphological criteria were considered as described in Table 2 [19].

### 2.4. Intravital Microscopy (IVM) of Mouse Colon

On day 7, half of the animals were anesthetized using sodium pentobarbital (90 mg/kg, 54 mg/mL; Ceva Sainte Animal, Montreal, QC, Canada) in a 1:2 dilution with 0.9% sodium chloride (NaCl). Depth of anesthesia was monitored using the pedal withdrawal reflex and additional anesthesia (9 mg/kg, 54 mg/mL; 1:10 dilution) was given as required. Fifteen minutes prior to imaging, Rhodamine-6G (0.05%, 1.5 mL/kg; Sigma-Aldrich, Oakville, ON, Canada) and fluorescein isothiocyanate-labelled bovine serum albumin (FITC-BSA, 5%, 1 mL/kg; Cat # A9771, Sigma-Aldrich, Oakville, ON, Canada) were administered via tail vein injection. A laparotomy was performed to expose the distal colon. Each mouse was placed on its side with the distal colon placed on the viewing platform of a specifically designed stage fixed to a heating pad [20]. A glass slide was placed over the intestine. A continuous flow of 0.9% NaCl (heated to 37 °C, 7 mL/hr) was administered to maintain physiological conditions. An epifluorescence microscope (Leica DMLM, Wetzlar, Germany) with a mercury-arc light source (LEJ EBQ 100; Carl Zeiss, Jena, Germany) enabled visualization of the intestinal microcirculation. Leukocyte trafficking was visualized within both submucosal collecting venules (V1, diameter: 50–100 μm) and post-capillary venules (V3, diameter: 20–35 μm). For each venule type, 6 videos of 30 s in length were acquired. Videos were analyzed in a blinded manual fashion using Fiji [21]. Adherent leukocytes were defined as labelled cells that remained immobile for the entire 30 s recording period. Endothelial surface area was estimated under the assumption of cylindrical vessel geometry. Non-adhesive and adhesive leukocytes were quantified and reported in cells/mm^2^ units.

### 2.5. Statistical Analysis

All data were analyzed using the statistical software package Prism 9 (GraphPad Software, La Jolla, CA, USA). Shapiro–Wilk test was used to confirm normal distribution of data. One-way ANOVA followed by Holm–Šídák’s multiple comparison test was used to analyze normally distributed data. Data were expressed as means ± standard deviations. 

## 3. Results

### 3.1. Mice Administered Dextran Sodium Sulfate (DSS) Exhibit Clinical Signs of Ulcerative Colitis 

**Figure 1:**
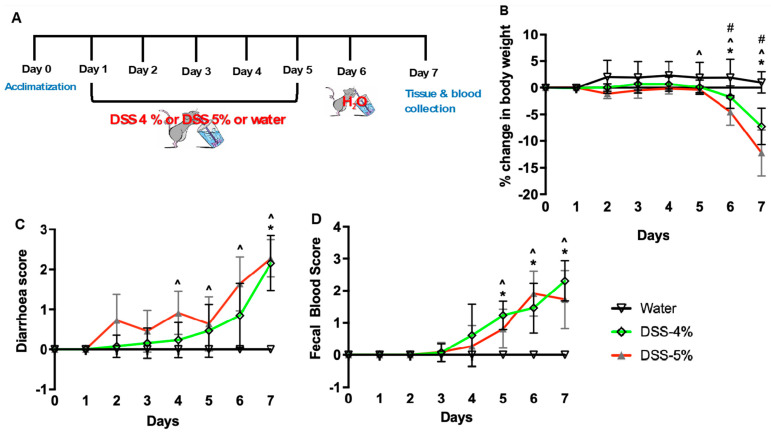
Mice administered dextran sodium sulfate (DSS) exhibit clinical signs of ulcerative colitis. (**A**) Schematic diagram of DSS-induced colitis mouse model. (**B**) Percentage reductions in body weight, (**C**) diarrhea score (stool consistency), and (**D**) fecal blood score in mice given either water or DSS 4% or DSS 5% from day 1 to day 5. Data are represented as means ± SEMs; N = 9 or 10. Statistical analysis was performed separately for each day using one-way ANOVA; * *p* < 0.05 water vs. DSS 4%; ^ *p* < 0.05 water vs. DSS 5%; # *p* < 0.05 DSS 4% vs. DSS 5%.

The DSS-induced colitis mouse model is commonly used to address the pathogenesis of inflammatory bowel disease (IBD) and to test the efficacy of small molecule compounds [22]. DSS is a synthetic sulphated polysaccharide that reproducibly induces acute and chronic colitis by interfering with the intestinal epithelial cell barrier, resulting in the release of cytokines and other inflammatory mediators [23,24]. In the current study, we induced experimental colitis in C57BL/6J mice by adding 4% or 5% DSS to the drinking water for 5 days (Figure 1A). Control mice were given unsupplemented water. Significant weight loss was evident in mice on day 6 and 7 after 4% or 5% DSS administration, respectively, compared to the control mice (Figure 1B). Furthermore, the weight loss was more severe in mice after 6 and 7 days of 5% DSS compared to the mice administered 4% DSS. To further determine the severity of the murine model of DSS-induced colitis, we assessed the clinical illness score by examining the stool consistency and the presence of blood in the stool. A higher score indicated a more severe disease condition. Both diarrhea score and fecal blood score remained 0 throughout the experiment in the mice given water (Figure 1C,D). In mice administered 4% DSS, the diarrhea score was significantly increased on day 7 whereas the presence of blood in the stool was observed only in DSS mice from Day 5–7 (Figure 1C,D). On the other hand, the diarrhea score was significantly increased as early at day 4 post DSS 5% administration, which was further associated with the presence of blood in the stool when compared to the mice administered water (Figure 1C,D). We also observed increased mortality (40%) in mice given 5% DSS. These mice were moribund and anemic and exhibited enlarged caeca. Together, these data suggest that mice administered 5% DSS exhibited a more severe phenotype of colitis that was associated with significant weight loss and increased clinical illness scores compared to the mice given either 4% DSS or water. 

### 3.2. DSS Administration Decreases Colon Length and Increases Colon Weight 

**Figure 2:**
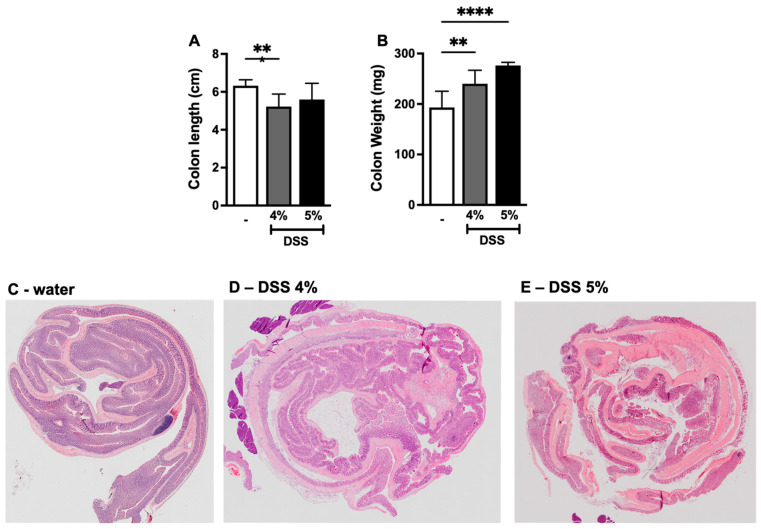
DSS administration decreases colon length and increases colon weight. (**A**) Colon length and (**B**) colon weight from mice given either water or DSS 4% or DSS 5%. Hematoxyline and eosin staining of colon tissues of mice administered either (**C**) water, (**D**) DSS 4%, or (**E**) DSS 5%. Data are represented as means ± SEMs; N = 8–10 (water and DSS 4%) and 5 (DSS 5%). White bar indicates mice given water only. ** *p* < 0.01, and **** *p* < 0.0001 using one-way ANOVA with a post hoc analysis.

**Figure 3:**
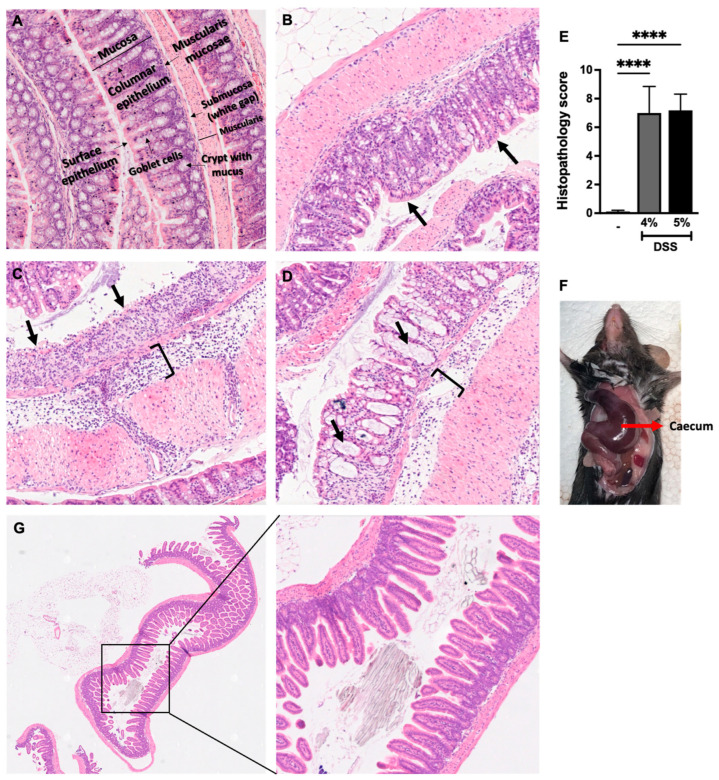
DSS administration induces histological alterations in mouse colons: Hematoxyline and eosin staining of colon tissues of mice given either water or DSS 4% or DSS 5%. (**A**) Well-defined intact layers of colon tissue from mice given water. Mice being given DSS 4% or DSS 5% causes histological alteration in colon tissue including (**B**) inflammatory cell infiltration with intact or undamaged surface epithelium (black arrow indicates intact surface epithelial layer); (**C**) inflammatory cell infiltration from mucosa to muscularis, surface epithelial erosion, and loss of crypts (black arrow indicates erosion of epithelial layer); and (**D**) crypts filled with mucus. The bracket “**]**” indicates the oedema in submucosa leading to the increase in the thickness of the colon tissue. (**E**) Histopathological score of colon tissues of mice given either water, DSS 4%, or DSS 5%. (**F**) A picture of a dissected mouse (administered DSS 5%) showing enlarged and discolored caecum. (**G**) Hematoxyline and eosin staining of small intestine in mice administered DSS 4%. Data are represented as means ± SEMs; N = 8–10 (water and DSS 4%) and 5 (DSS 5%). White bar indicates mice given water only. **** *p* < 0.0001 using one-way ANOVA with a post hoc analysis.

A shorter colon length and increased colon weight are considered as hallmarks of experimental colitis [18,25]. Our results revealed a significant reduction in colon length in mice given 4% DSS compared to the mice given water whereas no change in colon length was observed in mice given 5% DSS (Figure 2A). We next measured the colon weight and found that there was a significant increase in colon weight in mice administered either 4% or 5% DSS compared with the control mice (Figure 2B). Additionally, colonic inflammation was also examined through histology, where mouse colon tissues were stained with H&E. The histopathological scoring system was established based on criteria such as inflammatory cell infiltrates, epithelial changes, the extent of inflammation from mucosa to muscularis, and the mucosal architecture (Table 2). We noted that the appearance of the structure of the colon tissues from mice given 4% or 5% DSS was altered when compared with mice given just water (Figure 2C–E). In control mice, the layers of colon tissues such as the surface epithelium, mucosa, submucosa, and muscularis were well defined and intact (Figure 3A). On the other hand, a series of pathological changes were observed in DSS (4% and 5%)-group mice (Figure 3B–D). First, we noted the infiltration of inflammatory cells (with undamaged surface epithelium), which comprise of neutrophils, eosinophils, monocytes, plasma cells, and lymphocytes at differing ratios (Figure 3B). Second, we observed the infiltration of inflammatory cells with epithelial erosion, which is the loss of the surface epithelium with underlying inflammation reaching the basement membrane. If the epithelial erosion exceeds the submucosa, then it is defined as comprising ulcerations (Figure 3C). Third, we noted the loss of a crypt or crypt filled with mucus (Figure 3C,D). Finally, we noted the increase in the thickness of the colon wall due to the presence of significant edema in the submucosa of colon tissue (Figure 3D). Mice given DSS 4% or 5% exhibited a significant increase in their histopathological score compared with the mice given water (Figure 3E). While there was no significant difference in the histopathological score of the colon tissue between mice given DSS 4% and DSS 5% (Figure 3E), we did observe an enlarged and discolored caecum in mice given 5% DSS compared to those given DSS 4%, likely suggesting that these mice presented a more severe phenotype of colitis (Figure 3F). This finding was consistent with the clinical illness score, where mice in the DSS 5% group exhibited more severe forms of clinical signs compared to the mice given DSS 4%. Unlike Crohn’s disease, ulcerative colitis causes mucosal inflammation and ulcers, which involve the entire colon and sometimes also affect the caecum but not the small intestine. To ensure that DSS-induced colitis in mice is only restricted to the colon, mouse small intestine sections were stained with H&E. We noted that the layers of the small intestine were very well defined and intact in mice administered 4% DSS compared to water-given control mice, suggesting that DSS-induced inflammation was only localized to the colon tissue (Figure 3G).

### 3.3. DSS Administration Increases Colonic Leukocyte Recruitment in Mice

**Figure 4:**
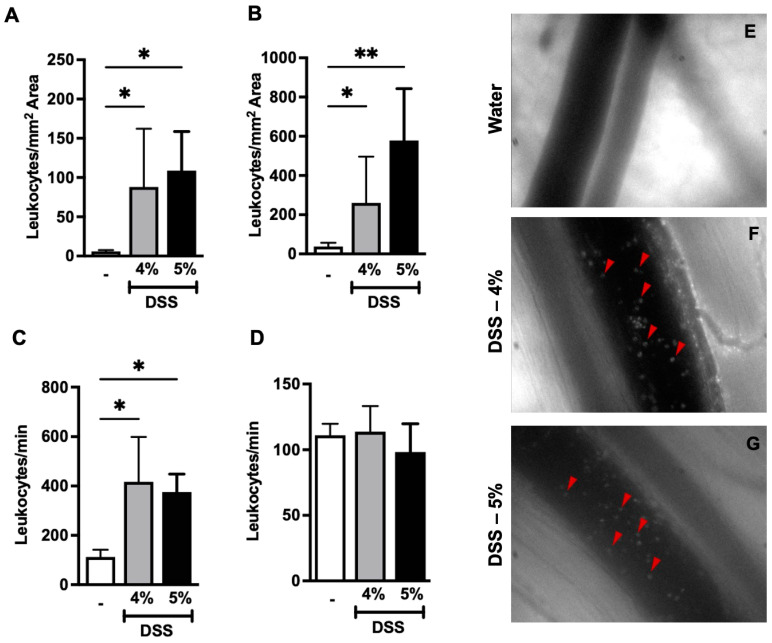
DSS administration increases leukocyte recruitment in colon tissue in mice. (**A**) Adherent and (**C**) non-adherent leukocytes from colon-collecting venules (V1) and (**E**–**G**) representative images of leukocytes (red triangles) in collecting venules of the colon from mice given either water, DSS 4%, or DSS 5%. (**B**) Adherent and (**D**) non-adherent leukocytes from post-capillary venules (V3) from mice given either water, DSS 4%, or DSS 5%. Data are represented as means ± SEMs; N = 8–10 (water and DSS 4%) and 5 (DSS 5%). White bar indicates mice on water. * *p* < 0.05 and ** *p* < 0.01 using one-way ANOVA with a post hoc analysis.

Higher leukocyte recruitment and increased leukocyte adhesion are characteristic of chronic inflammatory disorders [6,26]. In the current study, we employed intravital microscopy (IVM) for the experimental evaluation of the microcirculation of the colon. IVM allows the in vivo examination of many pathophysiological processes including leukocyte–endothelium interactions and capillary blood flow [27,28]. We noted that there was a significant increase in leukocyte adhesion in collecting colon submucosal venules (V1) (Figure 4A,E–G) and in post-capillary venules (V3) (Figure 4B) in mice given either 4% or 5% DSS compared to the mice given water. Furthermore, there was a significant increase in the number of rolling leukocytes in colon-collecting venules (V1) in mice administered either 4% or 5% DSS compared to the control group (Figure 4C,E–G), where no significant difference was observed in post-capillary venules among the groups (Figure 4D). These results imply that DSS administration (either 4% or 5%) amplifies the inflammatory phenotype by altering the colonic vasculature during experimentally induced ulcerative colitis in mice. Together, the results indicate that 4% DSS causes moderate inflammation whereas 5% DSS causes damage that may represent extensive irreversible tissue pathology. Given these observations, we decided to use only DSS 4% for our subsequent experiments. 

### 3.4. PEA Administration Ameliorates Clinical Signs of DSS-Induced Colitis in Mice

**Figure 5:**
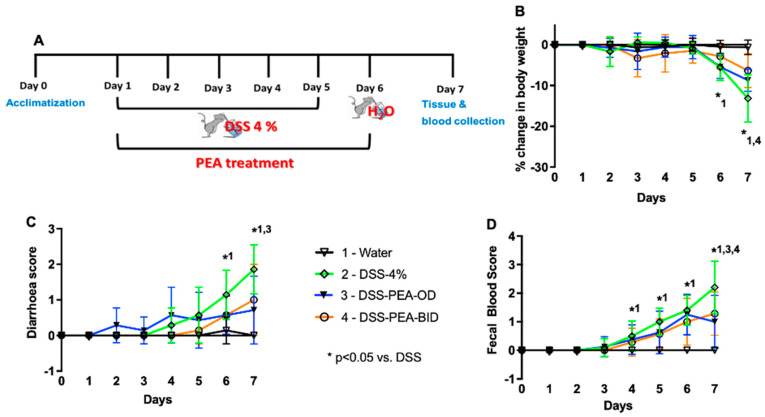
PEA treatment ameliorates clinical signs of DSS-induced colitis in mice. (**A**) Schematic diagram of DSS-induced colitis mouse model with PEA treatment. (**B**) Percentage reductions in body weight, (**C**) diarrhea score (stool consistency), and (**D**) fecal blood score in mice given either water or DSS 4% with or without PEA treatment (123 mg/kg, OD or BID, p.o.). Data are represented as means ± SEMs, N = 9 or 10. Statistical analysis was performed using two-way ANOVA: * *p* < 0.05 1-water vs. DSS 4%, * *p* < 0.05 3-DSS-PEA-OD vs. DSS 4%, and * *p* < 0.05 4-DSS-PEA-BID vs. DSS 4%.

We next examined the effect of PEA in an experimental-induced colitis model in mice. Mice given water or 4% DSS were treated with PEA (123 mg/kg p.o.) once or twice daily (Figure 5A). Based on the different doses of PEA employed in clinical trials, human-equivalent doses of PEA were chosen in the current study to examine its effect in DSS-induced colitis mice [29,30,31,32]. Since PEA has extremely low solubility, it was integrated into phytosome to increase its absorption and was delivered to mice through oral gavage for six days (Figure 5A). Additional water or DSS control groups were treated with PEA or empty phytosome, respectively (see Supplementary Figure S1). All water control groups did not show any clinical signs of colitis. In DSS control animals (with and without EP), we observed that mice exhibited the same reduction in body weight post 6 and 7 days of DSS administration (Supplementary Figure S1A). The reduction in body weight was also associated with an increased diarrhea score and fecal blood score in both DSS-alone and DSS + EP (all doses) groups (Supplementary Figure S1B,C). Therefore, we only compared the PEA treatment groups with the DSS control group without EP (Figure 5). We found that at day 6 and 7 following DSS administration, there was a significant reduction in body weight in mice given DSS alone compared to the control mice given water (Figure 5B). Furthermore, the DSS-induced reduction in body weight was ameliorated when DSS-administered mice were treated with PEA (BID) (Figure 5B). DSS-induced reduction in body weight was further associated with an increase in diarrhea score compared to the mice given water. This increase in diarrhea score was suppressed when DSS-administered mice were treated with PEA (OD) (Figure 5C). The diarrhea score in mice treated twice daily with PEA was also reduced (n.s.). There was a significant increase in fecal blood score from day 4 to 7 post DSS administration compared to the mice given water (Figure 5D). The increase in fecal blood score was attenuated when DSS-administered mice were treated with PEA (OD and BID) on day 7 (Figure 5D). Together, these data indicate that PEA treatment in mice attenuated DSS-induced clinical symptoms of ulcerative colitis.

### 3.5. PEA Treatment Did Not Attenuate DSS-Induced Decrease in Colon Length and Increase in Colon Weight

**Figure 6:**
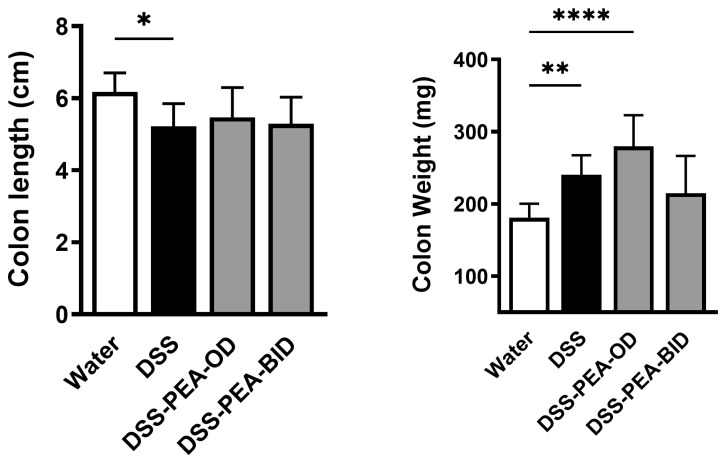
PEA administration partially reverses DSS-induced decrease in colon length. Colon length and colon weight from mice given either water or DSS 4% alone or DSS 4% treated with either PEA OD or BID. Data are represented as means ± SEMs; N = 5–6. * *p* < 0.05, ** *p* < 0.01, and **** *p* < 0.0001 using one-way ANOVA with a post hoc analysis.

Next, we examined the effect of PEA on colon length and weight. In the control experiments with water and DSS animals treated with EP, we did not see any effects on colon length and weight. Therefore, we used the water and DSS control groups without EP for comparisons again (Supplementary Figure S2A,B). We found that mice given DSS alone exhibited a significant reduction in colon length and increase in colon weight compared to control mice given water (Figure 6). In DSS mice treated with PEA, no change in colon length and weight was observed compared to untreated DSS animals (Figure 6). Together, these findings suggest that PEA treatment did not ameliorate DSS-induced decrease in colon length and increase in colon weight. 

### 3.6. PEA Treatment Partially Reverses the DSS-Induced Histopathological Changes in the Colon

**Figure 7:**
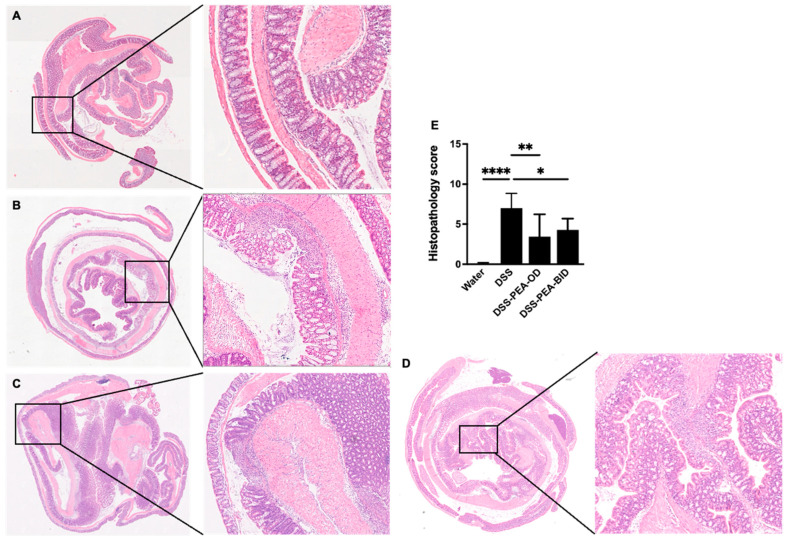
PEA treatment reduces histopathological alterations in mouse colons. Hematoxyline and eosin staining of colon tissues of mice administered either (**A**) water or (**B**) DSS 4% or DSS mice treated with (**C**) PEA, OD or (**D**) PEA, BID. (**E**) Histopathological scores of colon tissues of mice. Data are represented as means ± SEMs; N = 8–10. * *p* < 0.05, ** *p* < 0.01, and **** *p* < 0.0001 using one-way ANOVA with a post hoc analysis.

Surface epithelial erosion, the loss of crypts, and inflammation were absent in the water control group with PEA treatment (Figure 7A). In contrast, colon tissues from mice given 4% DSS alone showed extensive areas of mucosa with the loss of crypts or crypts filled with mucus, surface epithelial erosion, the infiltration of inflammatory cells, and ulceration (Figure 7B). We next examined the effect of PEA on the histopathology of mouse colons with DSS-mediated colitis. The treatment of PEA altered the appearance of colon tissue architecture, partially reverting it to being closer to the healthy control (water) group with reduced patches of inflammation and ulceration, epithelial erosion, and the loss of crypts (Figure 7C,D). The histopathological score was significantly (~7-fold) higher in the DSS group alone compared to the healthy water control group (Figure 7E). With PEA treatments, there was a significant reduction in the histopathological score compared to the mice administered DSS alone (Figure 7E). Overall, our results indicate that PEA treatment partially prevents DSS-mediated histopathological alterations in colon tissues in mice. 

### 3.7. PEA Treatment Reduces Leukocyte Recruitment in Mouse Colons in DSS-Induced Colitis

**Figure 8:**
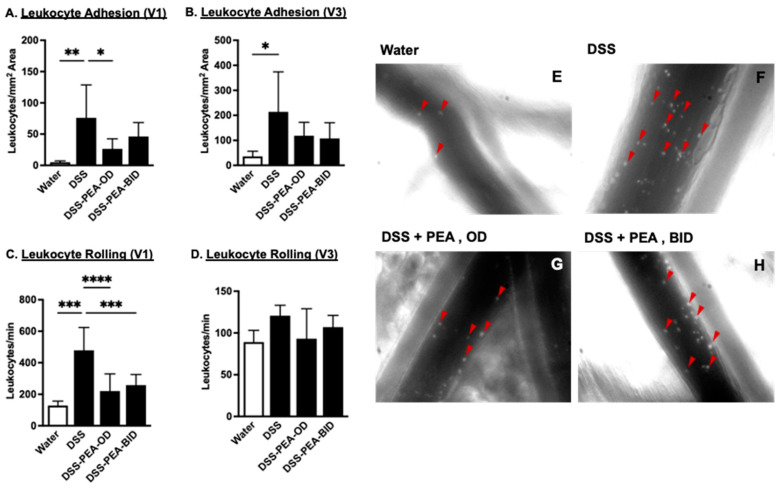
PEA treatment reduces DSS-induced increased in leukocyte recruitment in mouse colons. (**A**) Adherent and (**C**) non-adherent leukocytes and (**E**–**H**) representative images of leukocytes (red triangles) in collecting venules (V1) of the colon in mice given either water or DSS 4% and treated with PEA, OD or BID. (**B**) Adherent and (**D**) non-adherent leukocytes from post-capillary venules (V3) from mice given either water or DSS 4% or DSS 4% mice treated with PEA, OD or BID. Data are represented as means ± SEMs; N = 8–10. * *p* < 0.05, ** *p* < 0.01, *** *p* < 0.001, and **** *p* < 0.0001 using one-way ANOVA with a post hoc analysis.

We next examined whether PEA treatment alters colonic microcirculation in DSS-induced colitis. We noted that mice being treated with DSS alone elicited a significant increase in the number of adherent leukocytes in both V1 (Figure 8A,E–H)-collecting venules and V3 (Figure 8B) post-capillary venules and rolling leukocytes (only in V1 venules (Figure 8C) and not in V3 venules (Figure 8D)) compared to the control mice only given water. PEA treatment in DSS animals prevented the increase in the number of adherent leukocytes in V1 venules. Leukocyte adherence in V3 venules was reduced too but did not reach significance. The number of rolling leukocytes in V1 venules was suppressed when DSS-given mice were treated with PEA (Figure 8C); however, no changes were observed in rolling leukocytes in V3 venules when DSS mice were treated with PEA (Figure 8D). In conclusion, the DSS colitis-associated recruitment of both rolling and adherent leukocytes was partially attenuated by PEA treatment.

## 4. Discussion

PEA is a naturally occurring acylethanolamide and its anti-inflammatory, analgesic, and anti-convulsant properties are believed to be of potential therapeutic interest [33,34]. The present study was the first of its kind where PEA was phytosome-encapsulated and administered orally to examine its efficacy in treating DSS-induced ulcerative colitis. Prior studies had examined the effect of PEA on experimentally induced colitis in mice; however, the majority of these studies utilized the (1) intrarectal administration of DNBS to induce colitis in mice and (2) intraperitoneal route for PEA treatment in colitis mice [35,36,37], except one study wherein PEA was administered orally [38]. Our study employed DSS (given in drinking water) to induce colitis in mice due to its ease of administration, rapidity, reproducibility, and controllability [22]. Based on our pilot experiments, DSS 5% induced a very severe form of colitis compared to DSS 4% with significant mortality. Therefore, we decided to use only DSS 4% for our subsequent experiments. 

We first examined the effect of PEA on the clinical symptoms of DSS-induced colitis in mice. We observed that PEA (OD or BID) attenuated body weight reduction, diarrhea, and rectal bleeding. The shortening of the colon is a result of its thickening due to inflammation, oedema, and muscular hypertrophy [39,40]. These events are usually present in patients with UC, resulting in decreased transit times due to the shortening of the colon, likely contributing to diarrhea in UC patients [40]. In our experiments, we were able to reproduce the reduction of colon length through DSS administration. This DSS effect was not significant anymore following PEA treatment. This was in agreement with previous studies using parenteral PEA [35,37,38,41]. The effect on colon weight was inconclusive: PEA taken once daily increased colon weight whereas PEA taken twice daily seemed to reduce colon weight compared to untreated mice. However, the effect of PEA was further supported by its ability to reduce the histological signs of colon injury. One of the main pathological features of IBD is the infiltration of polymorphonuclear neutrophils and mononuclear cells into colonic tissues [19,40]. In line with the previous studies [11,37,38], PEA treatment resulted in the attenuation of intestinal inflammation, demonstrated by the reduction in oedema, infiltration of inflammatory cells, and ulcer formation. 

Immune system dysregulation and increased leukocyte recruitment are key pathological features of both human IBD and experimental colitis [7,40,42,43]. Using IVM to simultaneously monitor the trafficking of fluorescently labeled leukocytes in mice given DSS, our study was the first to examine the effect of PEA on intestinal microcirculation in DSS-induced colitis mice. Our in vivo study clearly demonstrated the activation of rolling and adherence of leukocytes in DSS-treated mouse colons. Interestingly, our study showed that PEA treatment (OD or BID) attenuated the increased leukocyte recruitment to the inflamed colonic venules, indicating a beneficial effect of PEA against the DSS-mediated amplification of the inflammatory response. Our findings are consistent with previous reports that also used IVM and noticed increased leukocyte recruitment in response to DSS administration in mouse colons [7,42,44]. These studies implicated leukocyte–platelet recruitment in colonic venules as requiring interaction between P-selectin and PSGL-1 on endothelial cells [7,44]. Meanwhile, another study revealed that nicotine treatment attenuates colitis through downregulating B2 integrin or MAdCAM-1 expression on the inflamed colonic microvessels [44]. Whether PEA suppresses leukocyte recruitment by modulating P-selectin/PSGL-1 interaction or MAdCAM-1 expression is not clear and warrants further investigation. Since the expression of cellular adhesion molecules such as selectins and integrins is dependent on inflammatory pathways, PEA action on anti-inflammatory receptors (e.g., CB2, PPARα) might play a role in this context. 

Our study was the first to investigate the efficacy of PEA encapsulated in phytosomes following oral administration in experimental ulcerative colitis. Here, we showed that PEA at a human-equivalent dose of 123 mg/kg (OD or BID) attenuated DSS-induced experimental colitis as represented by the reduction in the clinical signs of colitis, reduction in gross mucosal injury, and suppression of leukocyte recruitment at inflamed venules. These findings add to the growing body of data demonstrating the beneficial effects of PEA to control the acute phase of intestinal inflammation occurring during UC. PEA is a non-toxic endogenous lipid obtained from animal and vegetable foods, and due to its pharmacological and toxicological profile, PEA might be considered as a potential, easily manageable, and low-cost tool to treat colitis symptoms. Interestingly, PEA is currently administrated orally as a dietary supplement, in anti-inflammatory and analgesic preparations in dermatology and gynecology [45]. 

## 5. Conclusions and Future Direction

In conclusion, the current study suggested that PEA may represent a viable prophylactic and/or therapeutic option for patients with UC. However, it would be important to further elucidate whether anti-inflammatory effects of PEA are mediated by targeting CB1 or CB2 receptors, GPR55, PPAR alpha, or the TRPV1 channel. 

PEA signaling in vivo is regulated by the enzyme fatty acid amide hydrolase (FAAH) [25]; it would be of interest to assess whether the inhibition of FAAH potentiates the pharmacological effects of PEA. Collectively, our results suggest that orally administered, phytosome-encapsulated PEA has the potential to be used as a supplement to relieve symptoms in patients suffering from UC. 

## Figures and Tables

**Table 1:** Assessment of health, diarrhea, and fecal blood score.

Score	Diarrhea Score	Visible Fecal Blood Score	Eye Opening	Posture	Fur	Motor
0	Normal pellets	No blood	Open	No hunch	No change	Active
1	Slightly loose	Slightly bloody	Intermediate	mild hunching	Ruffled (Harsh)	No move for 2 s
2	Loose	Blood	Half	Moderate hunching	Piloerection	No move for 5 s
3	Very loose	Very bloody	Half + Discharge	Severe hunching	Ruffled/Pilo	No move for 10 s

Ruffled: not shiny, not groomed; piloerection: appearing of goosebumps on mice due to distress, ill health, or hypothermia; motor activity: exploring, moving, and grooming.

**Table 2:** Assessment of histopathological score.

Score	Extent of Inflammation	Inflammatory Cell Infiltration (Intact/Undamaged Epithelium)	Inflammatory Cell Infiltration (Epithelial Erosion)	Loss of Epithelium and Crypts, Crypts Filled with Mucus	Increased Space between MM and SM Due to Oedema
0	No evidence of inflammation				
1	Mucosa				
2	Mucosa + muscularis mucosae (MM)	Focal	Focal	Crypt filled with mucus	One-third
3	Mucosa + MM + submucosa (SM)	Multifocal	Multifocal	Epithelial erosion	Two-thirds
4	Mucosa + MM + SM + muscularis	Diffuse	Diffuse	Loss of crypt + ulceration	Full

## Data Availability

The raw data supporting the conclusions of this article will be made available by the authors on request.

## Supplementary Materials

The following supporting information can be downloaded at: https://www.mdpi.com/article/10.3390/biomedicines12051000/s1, Figure S1: Body weight and clinical scores in Water control groups and DSS groups with empty phytosome (EP); Figure S2: Colon length and colon weight scores in Water control groups and DSS groups with empty phytosome (EP); Figure S3: Intravital microscopy results in Water control groups and DSS groups with empty phytosome (EP).
