# Supplementary material for: Amelioration of Dextran Sodium Sulfate-Induced Colitis in Mice through Oral Administration of Palmitoylethanolamide
Source: Biomedicines. 2024 May 2;12(5):1000. doi: 10.3390/biomedicines12051000 (PMC11117589; doi:10.3390/biomedicines12051000)
Supplement: Supplementary file 1 [file biomedicines-12-01000-s001.zip › biomedicines-2957969-SI.pdf]

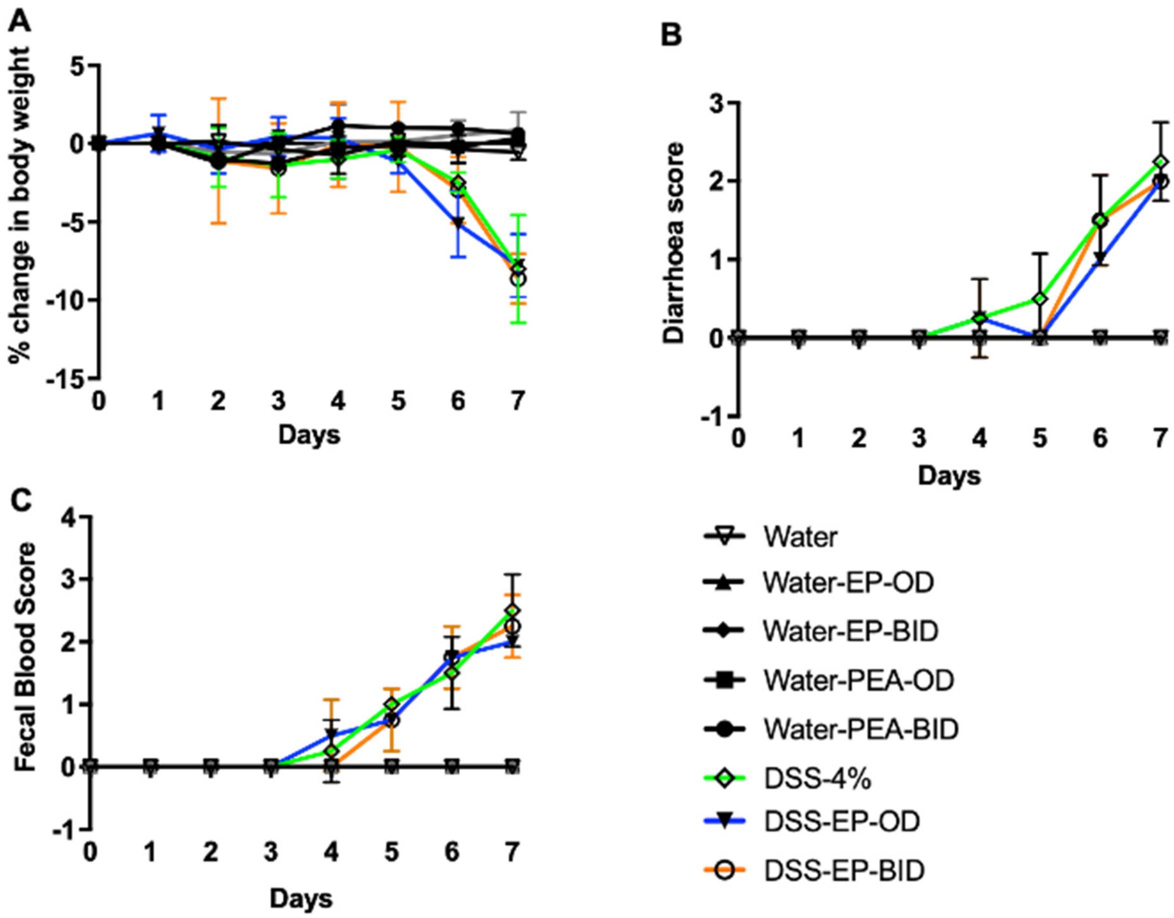

Sup. Figure S1: Body weight and clinical scores in Water control groups and DSS groups with empty phytosome (EP). (A) Percentage reduction in body weight, (B) Diarrhoea score (stool consistency) and (C) Fecal blood score of mice given either water or DSS 4% with or without empty phytosome (PE) treatment (123 mg/kg, OD or BID, p.o.) or with or without PEA (123 mg/kg, OD or BID, p.o.). Data represented as mean ± SEM, N = 8-10. Statistical analysis was performed using Two-Way ANOVA (not significant).

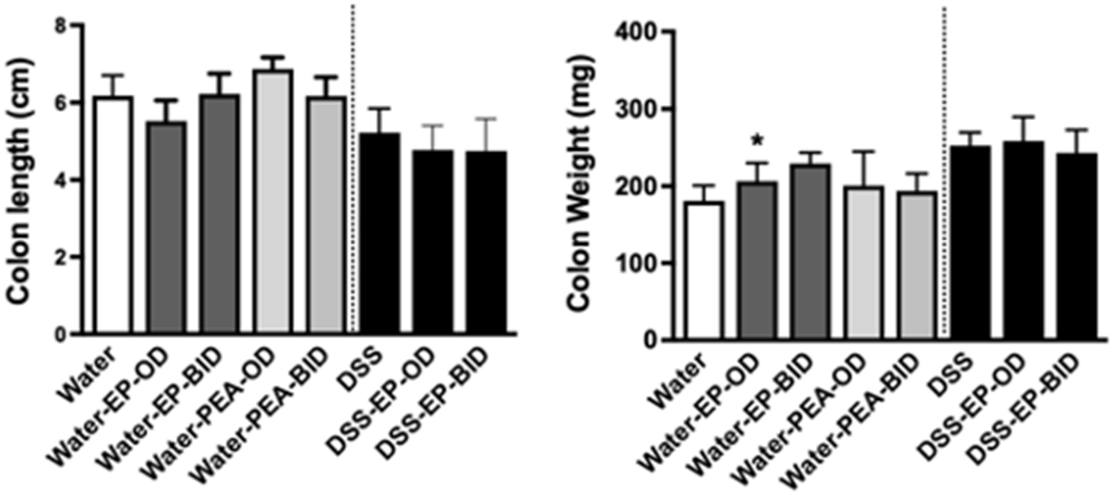

Sup. Figure S2: Colon length and colon weight scores in Water control groups and DSS groups with empty phytosome (EP). Data represented as mean ± SEM, N = 8-10. Statistical analysis was performed separately for each day using one-way Anova, *p<0.05 water vs water-EP-OD or -BID,

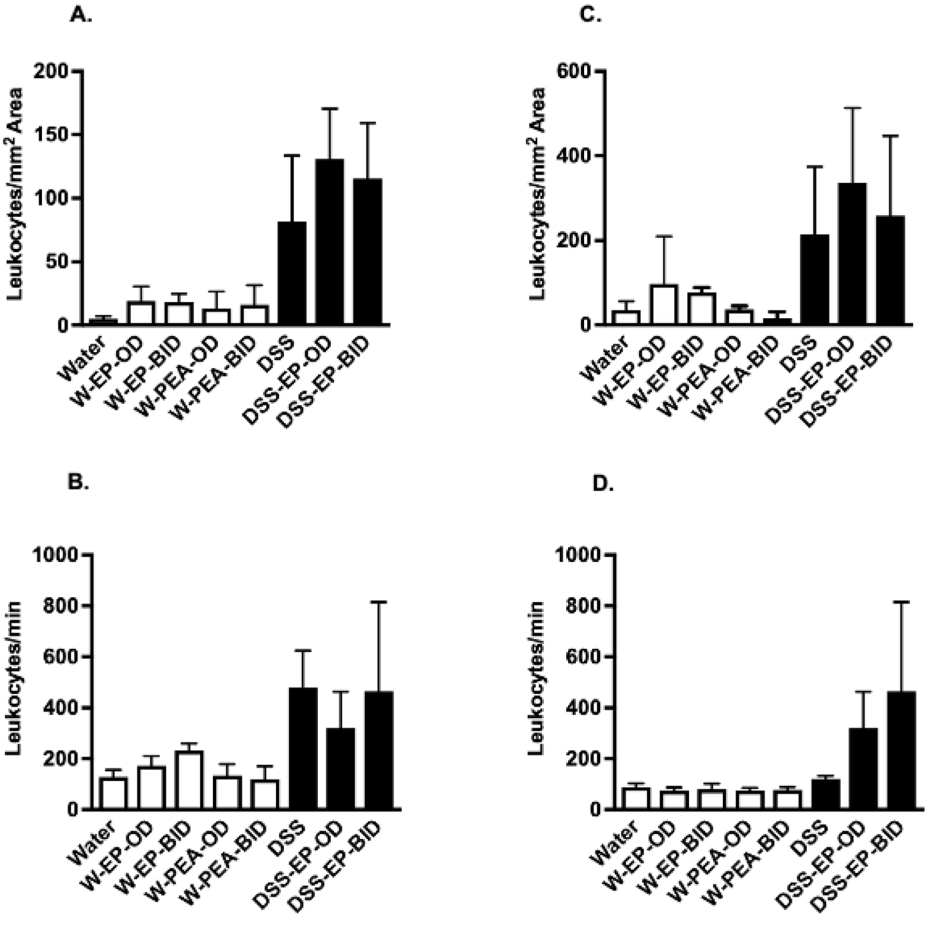

Sup. Figure S3: Intravital microscopy results in Water control groups and DSS groups with empty phytosome (EP). (A) Adherent and (B) non-adherent leukocytes from colon collecting venules (V1) and (C) Adherent and (D) non-adherent leukocyte from post-capillary venules (V3) from mice given either water or DSS 4% alone or DSS 4% treated with either PEA-OD or -BID or DSS 4% treated with EP-OD or -BID. Data represented as mean ± SEM, N = 8-10.
